# Peritoneal washing cytology status as a crucial prognostic determinant in patients with localized pancreatic ductal adenocarcinoma who underwent curative-intent resection following preoperative chemoradiotherapy

**DOI:** 10.1371/journal.pone.0309834

**Published:** 2024-09-06

**Authors:** Takuya Yuge, Yasuhiro Murata, Daisuke Noguchi, Takahiro Ito, Aoi Hayasaki, Yusuke Iizawa, Takehiro Fujii, Akihiro Tanemura, Naohisa Kuriyama, Masashi Kishiwada, Shugo Mizuno

**Affiliations:** Department of Hepatobiliary Pancreatic and Transplant Surgery, Mie University Graduate School of Medicine, Tsu, Mie, Japan; Affiliated Hospital of Nanjing University of Chinese Medicine: Jiangsu Province Academy of Traditional Chinese Medicine, CHINA

## Abstract

**Background:**

Prognostic implications of peritoneal washing cytology (CY) in patients with localized pancreatic ductal adenocarcinoma (PDAC) undergoing surgical resection following preoperative chemoradiotherapy (CRT) remain unclear. This study aimed to elucidate the prognostic significance and predictors of a positive CY status (CY+) after preoperative CRT.

**Methods:**

Clinical data from 141 patients with localized PDAC who underwent curative-intent resection after preoperative CRT were retrospectively analyzed to examine the association between CY+ and clinicopathological factors and survival.

**Results:**

CY+ was observed in six patients (4.3%). The CY+ group exhibited significantly higher preoperative serum levels of CA19-9 and a substantially greater incidence of tumor location in the pancreatic body or tail, along with pathological invasion to the anterior pancreatic capsule, than the CY− group. The CY+ group had a significantly higher incidence of peritoneal recurrence compared with the CY− group (83.3% vs. 18.5%, p = 0.002). Overall survival (OS) and recurrence-free survival (RFS) after surgery were significantly shorter in the CY+ group than in the CY− group (CY+ vs. CY−: 18.3 vs. 46.2 months, p = 0.001, and 8.9 vs. 17.7 months, p = 0.009, respectively). Multivariate analyses identified CY+ as an independent prognostic factor for worse OS (hazard ratio 5.00, 95% confidence interval 1.03–12.31) and RFS (hazard ratio 2.58, 95% confidence interval 1.04–6.43). Local invasion grade on imaging before CRT, limited histological response to CRT, and absence of adjuvant chemotherapy were independent predictors of worse OS and RFS.

**Conclusion:**

Despite the relatively low incidence of CY+ after preoperative CRT, it emerged as an independent prognostic factor in patients with localized PDAC undergoing curative-intent resection following preoperative CRT.

## Introduction

Pancreatic ductal adenocarcinoma (PDAC) presents a formidable challenge due to its aggressive nature and the limited range of available treatments. Although surgical resection is the primary method for achieving a cure in patients with PDAC, its prognosis remains unsatisfactory. Locoregional and distant recurrences, including peritoneal recurrence, are frequently observed even after curative-intent resection. In recent years, a multidisciplinary management approach, specifically preoperative systemic chemotherapy with or without concurrent radiotherapy followed by surgical evaluation, has emerged as a pivotal component in the treatment of localized PDAC, with the aim of improving patient outcomes [1].

Peritoneal washing cytology (CY) is routinely performed during staging laparoscopy or initial laparotomy to microscopically investigate disseminated cancer cells. Positive peritoneal washing cytology (CY+) is classified as distant metastasis (M1) by the Union for International Cancer Control tumor-node-metastasis classification system [2] and the American Joint Committee on Cancer [3]. Moreover, it is classified as M1 in the recently revised eighth edition of the Japan Pancreas Society classification [4].

The prognosis of individuals with confirmed CY+ has been firmly established to be exceedingly dismal among those with localized PDAC who undergo upfront surgery. Multiple meta-analyses have emerged, discouraging the prioritization of upfront surgery for localized patients with PDAC and documented CY+ status. Several meta-analyses have reported a significant association between CY+ and poor prognosis in resectable PDAC with surgical resection [5–7], whereas others have indicated a substantial correlation with peritoneal recurrence after surgery [8]. However, the limitations of studies on CY include their retrospective nature and the lack of prospective studies and randomized controlled trials investigating the prognostic significance of surgical resection in patients with PDAC and CY+ status. Consequently, the clinical role of the CY status in patients with PDAC undergoing surgical resection remains controversial. This is especially true in the current trend where surgical resection after multidisciplinary treatment, including preoperative chemotherapy or chemoradiotherapy (CRT), is becoming predominant in PDAC. The importance of surgical resection for patients with localized PDAC and confirmed CY+ following such preoperative therapies, and where the primary lesion is deemed surgically resectable, has not been sufficiently examined. Specifically, data regarding the prevalence of CY+ status and its effect on survival in patients with localized PDAC undergoing curative-intent resection following preoperative CRT are limited.

This study aimed to elucidate the frequency of CY+ and its prognostic significance in patients with localized PDAC who underwent curative-intent resection after preoperative CRT. It also aimed to identify preoperative risk factors that can predict the occurrence of CY+.

## Materials and methods

### Study design

**Fig 1:**
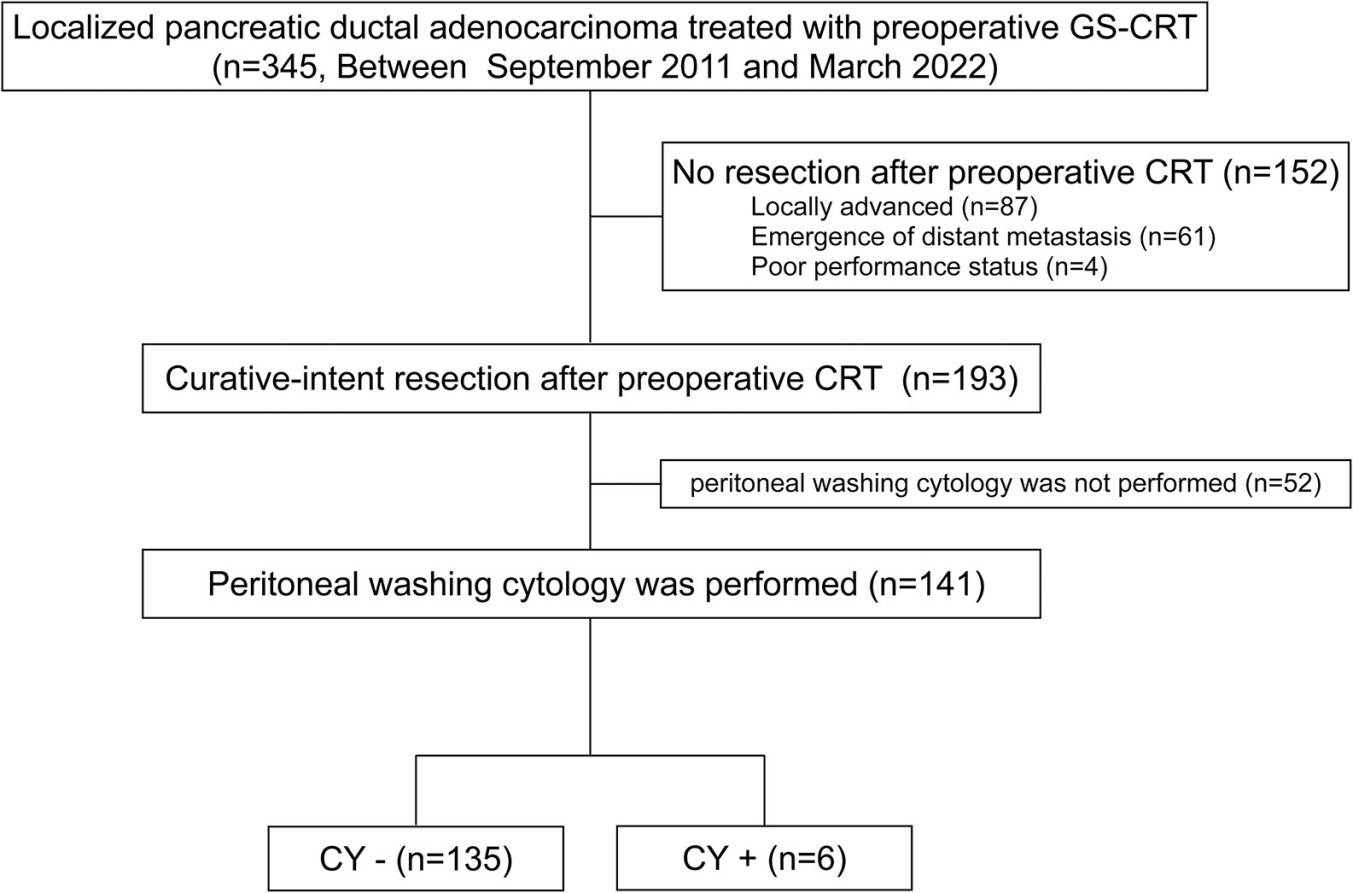
Selection of subjects and study design based on the results of peritoneal washing cytology for patients with localized pancreatic adenocarcinoma who underwent curative-intent resection after preoperative chemoradiotherapy.

This study was conducted ethically, adhering to the principles outlined in the Declaration of Helsinki, and received approval from by the ethics committee of Mie University Graduate School of Medicine (No. H2020-118). Informed Consent was waived due to the retrospective nature of the study. Data for this retrospective analysis were sourced from a prospectively maintained institutional database comprising patients with localized pancreatic ductal adenocarcinoma (PDAC) who underwent curative-intent resection following preoperative chemoradiotherapy (CRT) at the Department of Hepatobiliary Pancreatic and Transplant Surgery, Mie University Hospital, Mie, Japan. The data were accessed for research purposes in January 24th, 2024. Patient selection and study design are illustrated in Fig 1. The study included 345 consecutive patients diagnosed with cytologically proven localized PDAC, all of whom underwent curative-intent resection following preoperative CRT between September 2011 and March 2022. From the initial cohort, 152 patients were excluded. Exclusions were made on the basis of lack of resection following CRT, comprising 87 cases of locally advanced unresectable disease, 61 cases of distant metastasis, and 4 cases with poor performance status upon re-evaluation after CRT. Of the 193 patients who underwent resection following preoperative CRT, 52 were further excluded due to lack of intraoperative peritoneal washing. Consequently, the final analysis was conducted on the remaining 141 patients who underwent resection after preoperative CRT and had intraoperative CY performed. These patients were categorized into CY+ (n = 6) and CY− (n = 135) groups. The positive rate of peritoneal washing cytology was 4.3%. Pretreatment patient demographics and clinical outcomes, including surgical and pathological data, were compared between the two groups. The study compared the overall survival (OS) after initial treatment and the OS and recurrence-free survival (RFS) after surgery the groups. Univariate and multivariate COX regression analyses were conducted to identify clinicopathological factors significantly associated with OS and RFS after surgery. The day of the final follow-up was November 30, 2023. The median follow-up period after initial treatment was 33.8 months.

### Radiologic assessment of tumor resectability

Initial tumor staging and resectability were determined based on findings from pretreatment tetraphasic multidetector 64-row contrast-enhanced computed tomography (MD-CT) with thin slices at intervals of 1.00 mm, following a defined pancreatic protocol during the initial visit. In accordance with the seventh edition of the Japan Pancreas Society classification, local invasion grade, lymph node metastasis, and tumor resectability were evaluated based on MD-CT findings [9]. In terms of local invasion grade, the T category was defined as follows: T1, tumor limited to the pancreas with a size of ≤20 mm; T2, tumor limited to the pancreas exceeding 20 mm; T3, tumor extending beyond the pancreas but without involvement of the celiac axis (CA) or superior mesenteric artery (SMA); and T4, tumor involving the CA or SMA. Additionally, local invasion factors, including bile duct invasion, duodenal invasion, invasion of the serosal side of the anterior pancreatic tissue, retropancreatic tissue invasion, portal venous system invasion, arterial system invasion, extrapancreatic nerve plexus invasion, and invasion of other organs, were evaluated. Localized PDAC was classified into four anatomical resectability categories: resectable, borderline resectable with superior mesenteric vein or portal vein invasion alone, borderline resectable with arterial contact, and locally advanced unresectable, as previously described. Patients with metastatic PDAC were systematically excluded from the CRT treatment protocol. Staging laparoscopy with peritoneal washing CY before initiating preoperative CRT has never been performed in eligible patients.

### Measurement of peritoneal washing CY

Peritoneal washing CY was performed after laparotomy and before pancreatectomy, according to the Japanese General Rules for the Study of Pancreatic Cancer [9]. Briefly, 100 ml of physiological saline solution was introduced into the pelvic cavity immediately after laparotomy. After gentle washing, peritoneal washing fluids were collected from the pouch of Douglas using a catheter and syringe. The collected fluid was then sent to the Department of Pathology for a permanent cytological examination. Smears were prepared from the centrifuged deposits using Papanicolaou and May Giemsa staining and examined by experienced pathologists. In the present study, CY+ was defined as the presence of malignant cells in peritoneal washings or atypical cells that were highly suggestive of malignancy. Patients were classified into two groups based on the final cytological diagnosis of CY, which typically became evident a few days after surgery. In the present study, intraoperative diagnosis of CY was not performed in all eligible patients.

### Treatment protocol for preoperative CRT, surgical procedures, and adjuvant chemotherapy

All 141 patients received preoperative S-1 plus gemcitabine-based preoperative CRT, as previously reported [10]. Patients received oral administration of S-1 twice daily at a dosage of 60 mg/m^2^ per day on days 1 to 21 of a 28-day cycle for two cycles. Additionally, they received gemcitabine infusion at a dosage of 600 mg/m^2^ on days 8, 22, 36, and 50. Three-dimensional conformal radiotherapy was administered concurrently, as previously described. The total radiation dose ranged from 45 to 50.4 Gy, delivered in 25 to 28 fractions. Following CRT completion, all patients underwent resectability reassessment and staging evaluation by MD-CT 4–6 weeks post-CRT. Surgery was indicated for patients showing local downstaging (e.g., from locally advanced unresectable to borderline resectable) or those with no encasement or deformity of major arteries such as the CA, SMA, or jejunal arteries, as previously reported. Additionally, either normalization or a remarkable decrease in serum CA19-9 levels served as the criterion for surgical decision-making. Patients deemed unsuitable for surgery continued receiving additional chemotherapy (gemcitabine plus S1 Data set) and underwent re-evaluation by MD-CT, with surgery planned if it was considered an indication during re-evaluation.

During the laparotomy, pancreatectomy with regional lymph node dissection was performed in patients without distant metastases. Pancreaticoduodenectomy was performed using an anterior approach to ensure a negative surgical margin around the SMA. Distal pancreatectomy was performed using radical antegrade modular pancreatosplenectomy. Resection and reconstruction of the portal and superior mesenteric veins were performed when the separation of the pancreatic head from these vessels without leaving a gross tumor on the vessel was not possible. Segmental arterial resection and reconstruction were performed in selected patients with limited involvement of the common hepatic artery. With regard to the degree of residual tumor, surgery with a microscopically negative margin was classified as an R0 resection, whereas surgery with a microscopically positive margin was classified as an R1 resection. In accordance with the JPS classification system, the pathological degree of local invasion and histological response to preoperative CRT were evaluated [9]. The estimated rate (%) of residual tumor was defined as the volume of cancer cells considered viable divided by the estimated tumor volume before treatment. The grading of histological response is shown based on the estimated rate of residual tumor as follows: grade 1 (poor response), 50% or more (1a: 90% or more, 1b: 50% or more and less than 90%); grade 2 (moderate response), 10% or more and less than 50%; grade 3 (marked response), less than 10%; and grade 4 (complete response), no viable cancer cells were present. In this study, a histological response graded 3 or 4 was considered a marked response, whereas a response graded 1 or 2 was considered a limited response [11]. Postoperative complications were graded according to the Clavien–Dindo classification [12]. Based on previous evidence supporting the efficacy of adjuvant chemotherapy, either S-1 or gemcitabine chemotherapy was routinely administered during the postoperative period [13,14].

All patients underwent regular evaluations, including monthly physical examinations, laboratory tests, including serum levels of CA19-9 every 2 or 3 months, and MDCT every 3 months within the first 2 years, followed by assessments every 6 months after surgery. In instances where the serum levels of tumor markers increased, further evaluation using MDCT was conducted. Recurrent disease sites were documented at the time of initial recurrence.

### Statistical analyses

Continuous and categorical variables were summarized as median (interquartile range) and frequency, respectively, in each group. Categorical variables were compared using either the Fisher’s exact test or chi-square test, and continuous variables were compared using the Mann–Whitney U test between the groups. OS was defined as the time interval from the initial treatment to the date of death or last follow-up. RFS was defined as the time from the surgical resection to the date of disease recurrence or death. Patients who were alive at the time of their last disease evaluation were censored for the OS analysis. Survival curves were constructed using the Kaplan–Meier method and compared between groups using the log-rank test. Statistical analyses of the survival curves were performed using the Cox proportional hazards model, and hazard ratios (HR) and 95% confidence intervals (CI) were calculated. Associations between OS and RFS after surgery and potential prognostic factors were assessed using the log-rank test in the univariate analysis. The multivariate Cox proportional hazards model was applied by including the significant variables in the initial log-rank test. All statistical analyses were performed using JMP Pro software (SAS Institute Inc., Cary, NC, USA). Statistical significance was set at p<0.05.

## Results

### Comparison of preoperative patient demographics between patients with CY+ and patients with CY−

A comparison of patient demographics based on the CY status is presented in Table 1. In all patients, the median age was 68 (61–73) years, and the tumor locations were identified as the head in 106 patients and the body or tail in 35 patients. Initial tumor resectability was classified as resectable (R) in 45 patients, borderline resectable in 60, and locally advanced unresectable in 36. When comparing based on CY status, the incidence of tumor location in the body or tail was significantly higher in the CY+ group than in the CY− group (66.7% vs. 23.0%, p = 0.033). Regarding serum tumor markers, the serum level of CA19-9 before surgery was significantly higher in the CY+ group compared to the CY− group (66.9 vs. 22.2 U/mL, p = 0.038). Other factors, including patient conditional factors such as the neutrophil to lymphocyte ratio and the prognostic nutritional index before CRT and surgery, as well as the grade of local invasion, and initial resectability as assessed by CT scans before CRT and surgery, did not show significant differences between the two groups.

**Table 1:** Comparison of the patient preoperative demographics based on the results of peritoneal washing cytology (n = 141).

variables	All cases (n = 141)	CY + (n = 6)	CY—(n = 135)	*p* value
Age	68 (61–73)	71 (69–80)	68 (60–73)	0.071
Gender (male/female)	87/54	5/1	82/53	0.407
Performance status (0/1/2)	109/28/4	4/1/1	105/27/3	0.114
Laboratory data				
Neutrophil to lymphocyte ratio before CRT	2.2 (1.3–3.3)	3.6 (1.7–4.6)	2.4 (1.7–3.5)	0.292
Prognostic nutritional index before CRT	47.7 (43.8–52.1)	47.3 (44.5–49.2)	47.9 (43.7–52.2)	0.425
Neutrophil to lymphocyte ratio before surgery	3.6 (2.6–5)	2.9 (1.8–3.7)	3.7 (2.6–5.2)	0.100
Prognostic nutritional index before surgery	42.7 (40.1–45.4)	44.4 (41.5–47.9)	42.7 (40.1–45.2)	0.263
Serum level of CEA before CRT (ng/mL)	3.1 (2.0–5.8)	5.0 (2.0–5.6)	3.2 (2.8–8.5)	0.394
Serum level CA19-9 before CRT (U/mL)	147.5 (39.2–568.5)	459.5 (298.1–1754.7)	128.7 (37–478.6)	0.092
Serum level of CEA before surgery (ng/mL)	2.9 (1.9–4.5)	3.8 (2.2–24)	2.9 (1.9–4.5)	0.250
Serum level of CA19-9 before surgery (U/mL)	24.5 (10.0–61.0)	66.9 (32.1–130.9)	22.2 (9.8–58.6)	**0.038**
Imaging parameters				
Tumor location (head/body or tail)	106/35	2/4	104/31	**0.033**
Tumor size before CRT (mm)	29.0 (24.3–36.4)	23.3 (18.5–40)	29 (24.4–36.4)	0.456
Tumor size before surgery (mm)	24.8 (19.4–31.3)	21.8 (16.7–40.7)	25 (19.6–31.1)	0.922
Grade of local invasion before CRT (T1/T2/T3/T4)	2/0/75/64	0/0/3/3	2/0/72/61	0.898
Bile duct invasion before CRT (no/yes)	68/73	4/2	64/71	0.429
Duodenal invasion before CRT (no/yes)	88/53	4/2	84/51	1.000
Anterior pancreatic tissue invasion before CRT (no/yes)	33/108	0/6	33/102	0.336
Retropancreatic tissue invasion before CRT (no/yes)	27/114	0/6	27/108	0.595
Portal venous system invasion before CRT (no/yes)	21/120	1/5	20/115	1.000
CA invasion before CRT (no/yes)	124/17	5/1	119/16	0.544
SMA invasion before CRT (no/yes)	81/60	3/3	78/57	0.700
CHA invasion before CRT (no/yes)	108/33	5/1	103/32	1.000
Extrapancreatic nerve plexus invasion before CRT (no/yes)	62/79	3/3	59/76	1.000
Invasion of other organs before CRT (no/yes)	118/23	4/2	114/21	0.253
Grade of local invasion before surgery (T1/T2/T3/T4)	2/0/78/61	0/2/4	2/0/76/57	0.474
Lymph nodal metastasis before CRT (cN0/N1a/N1b)	120/17/4	5/1/0	115/16/4	0.865
Lymph nodal metastasis before surgery (cN0/N1a/N1b)	131/7/3	6/0/0	125/7/3	0.637
Resectability before CRT (R/BR-PV/BR-A/UR-LA)	45/23/37/36	3/0/2/1	42/23/35/35	0.577
Resectability before surgery (R/BR-PV/BR-A/UR-LA)	52/19/31/39	2/0/3/1	49/19/28/38	0.302

CRT; chemoradiotherapy, CA; celiac axis, SMA; superior mesenteric artery, CHA; common hepatic artery, R; resectable, BR; borderline resectable, UR-LA; locally advanced unresectable.

### Comparison of surgical and pathological outcomes between patients with CY+ and patients with CY−

The comparison of the surgical and pathological outcomes based on CY status is shown in Table 2. In all patients, the median interval from the initial treatment to surgery was 4.4 (3.6–6.7) months. Pancreaticoduodenectomy was performed in 120 patients, while distal pancreatectomy was carried out in 21 patients. The median operation time was 518 (432–612) minutes, and the median estimated blood loss was 597 (349–1137) ml. Forty-one patients (29.1%) experienced major (Clavien–Dindo IIIa or higher) postoperative complications. Subsequent to surgery, 117 patients (83.0%) completed adjuvant chemotherapy. The interval from the initial treatment to surgery did not exhibit a statistically significant difference between the two groups (CY+ vs. CY−: 4.7 vs. 4.3 months). When comparing surgical outcomes based on CY status, the operation time was significantly shorter in the CY+ group than in the CY− group (450 vs. 519 minutes, p = 0.014). The incidence of major postoperative complications did not significantly differ between the two groups. No significant difference was found in the frequency of adjuvant chemotherapy between the two groups. Regarding the pathological results, the grading of local invasion identified extrapancreatic invasion (pT3) in 95 patients and the absence of extrapancreatic invasion (pT1 or pT2) in 46 patients. R0 resection was achieved in 128 patients (90.8%), and a major histological response (grades 3–4) was observed in 63 patients (44.7%). When comparing pathological results, the incidence of anterior pancreatic tissue invasion on the serosal side was significantly higher in the CY+ group than in the CY− group (66.7% vs. 24.4%, p = 0.041). However, other pathological parameters, including the pathological grading of local invasion, rates of pathological lymph nodal metastasis, R0 resection, and major histological response to CRT (grade 3 or more), did not significantly differ between the two groups. With regard to the initial recurrence site, CY+ demonstrated a significantly higher incidence of peritoneal recurrence compared with CY− (83.3% vs. 18.5%, p = 0.002).

**Table 2:** Comparison of the surgical outcomes and mode of recurrence based on the results of peritoneal washing cytology (n = 141).

variables	All cases (n = 141)	CY + (n = 6)	CY—(n = 135)	*p* value
Surgical outcomes				
Interval from the initial treatment to surgery (months)	4.4 (3.6–6.7)	4.7 (3.1–5.0)	4.3 (3.6–6.9)	0.394
Surgical procedure (PD/DP)	120/21	5/1	115/20	1.000
Operation time (min)	518 (432–612)	450 (380–506)	519 (432–611)	**0.014**
Estimated blood loss (ml)	597 (349–1137)	486 (319–856)	593 (331–1141)	0.414
Major postoperative complications* (yes/no)	41/100	1/5	40/95	0.672
Conducting adjuvant chemotherapy (yes/no)	117/24	4/2	113/22	0.270
Pathological parameters				
Grade of local invasion (pT1 or T2/T3)	46/95	1/5	45/90	0.219
Pathological lymph node metastasis (negative/positive)	110/31	6/0	104/31	0.384
Bile duct invasion (no/yes)	115/26	6/0	109/26	0.287
Duodenal invasion (no/yes)	103/38	5/1	98/37	0.485
Serosal side of the anterior pancreatic tissue invasion (no/yes)	104/37	4/2	33/102	**0.041**
Retropancreatic tissue invasion (no/yes)	75/66	1/5	74/61	0.078
Portal venous system invasion (no/yes)	111/30	5/1	106/29	0.623
Extrapancreatic nerve plexus invasion (no/yes)	103/38	3/3	100/35	0.197
Invasion of other organs (no/yes)	139/2	6/0	133/2	0.916
Degree of the residual tumor (R0/R1)	128/13	6/0	122/13	0.554
Histological response to CRT (grade1-2/3-4)	78/63	3/3	75/60	1.000
Mode of recurrence				
Liver (yes/no)	22/119	2/4	20/115	0.236
Peritoneal (yes/no)	30/111	5/1	25/110	**0.002**
Lymph node (yes/no)	5/136	0/6	5/130	1.000
Lung (yes/no)	26/115	0/6	26/109	0.593
Local (yes/no)	10/131	1/5	9/126	0.362

CRT; chemoradiotherapy, CEA; carcinoembryonic antigen, CA; carbohydrate antigen, R; resectable, BR; borderline resectable, UR-LA; locally advanced unresectable.

### Survival analyses of clinicopathological factors in all eligible patients

**Fig 2:**
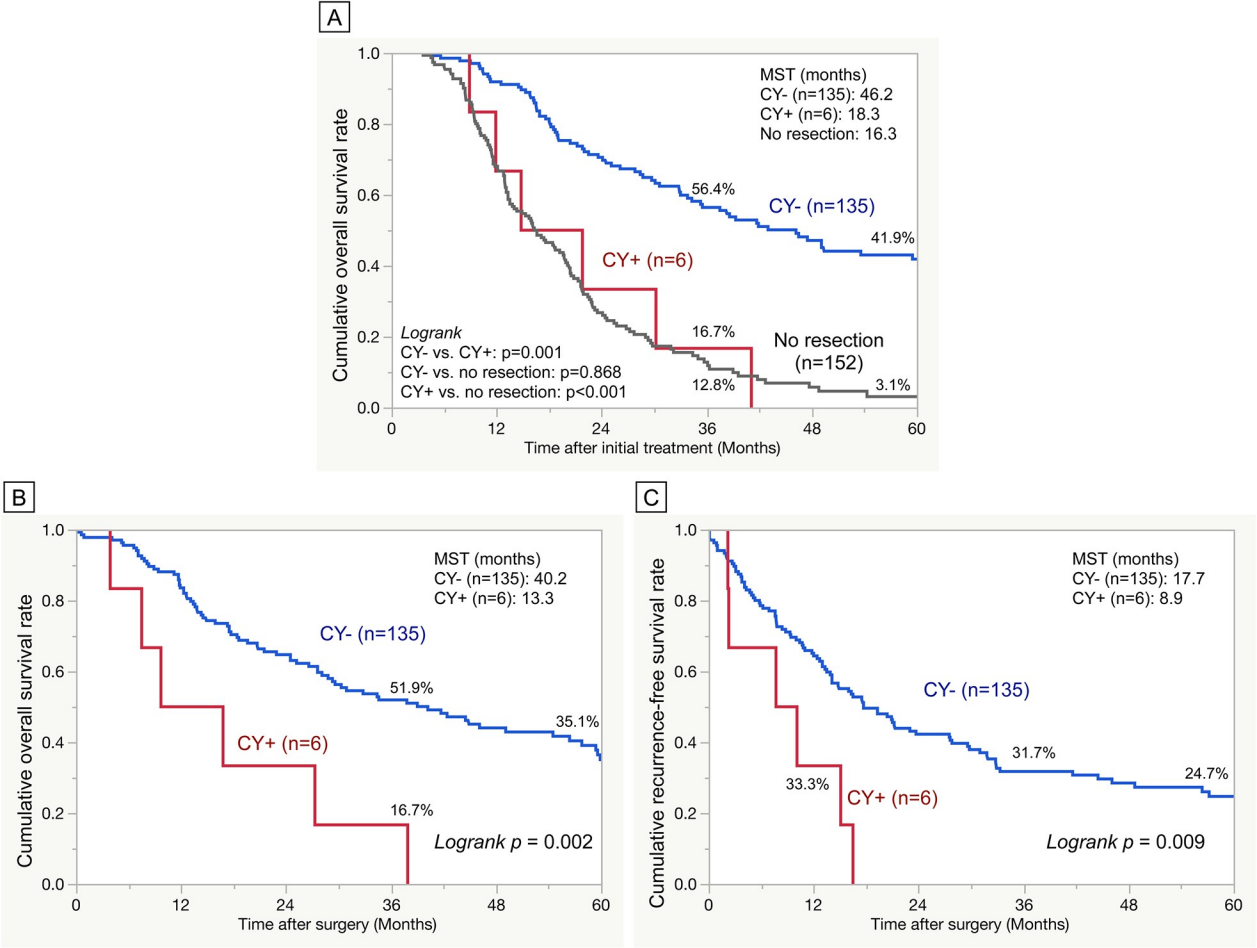
Overall survival and recurrence-free survival curves based on the results of peritoneal washing cytology. A. Overall survival rate after the initial treatment for CY− (n = 135) vs. CY+ (n = 6) vs. no resection cases after CRT (n = 152). B. Overall survival rate after surgery for CY− vs. CY+. C. Recurrence-free survival rate after surgery for CY− vs. CY+.

The OS after initial treatment was significantly shorter in the CY+ group than in the CY− group (CY+ vs. CY−: 18.3 vs. 46.2 months, p = 0.001). However, no significant difference in OS after initial treatment was observed between the CY+ group and the cohort with no resection after CRT (CY+ vs. no resection: 18.3 vs. 16.3 months, p = 0.868; Fig 2A). Similarly, the CY+ group significantly exhibited shorter OS after surgery than the CY− group (CY+ vs. CY−: 13.3 vs. 40.2 months, p = 0.002; Fig 2B). Furthermore, the CY+ group demonstrated significantly shorter RFS than the CY− group (CY+ vs. CY−: 8.9 vs. 17.7 months, p = 0.009; Fig 2C). The results of the univariate and multivariate analyses of the clinicopathological factors associated with OS and RFS after surgery are presented in Table 3. In the univariate analysis of OS after surgery, factors such as the grade of local invasion before CRT on CT, estimated operative blood loss, major postoperative complications, administration of adjuvant chemotherapy, pathological grade of local invasion, extrapancreatic nerve plexus invasion, degree of residual tumor, histological response to CRT, and CY status were identified as significant factors for OS after surgery. Multivariate analysis revealed that tumor involving CA or SMA (cT4) before CRT on CT scans, estimated operative blood loss (×10^2^ ml), the absence of adjuvant chemotherapy, limited histological response to CRT (grades 1–2), and CY+ were independent prognostic factors significantly associated with worse OS after surgery (HR (95% CI): 1.66 (1.01–2.73), 1.02 (1.00–1.03), 3.99 (2.23–7.13), 2.10 (1.29–3.43), and 5.00 (2.03–12.31), respectively). In the univariate analysis of RFS after surgery, the significant factors included the grade of local invasion before CRT on CT scans, estimated operative blood loss, administration of adjuvant chemotherapy, pathological grade of local invasion, degree of residual tumor, histological response to CRT, and CY status. The multivariate analysis revealed that tumors involving CA or SMA (cT4) before CRT on CT scans, the absence of adjuvant chemotherapy, presence of pathological extrapancreatic invasion (pT3), limited histological response to CRT (grades 1–2), and CY+ were independent prognostic factors significantly associated with worse RFS (HR (95% CI): 1.57 (1.03–2.39), 2.04 (1.18–3.52), 1.69 (1.04–2.72), 1.72 (1.10–2.70), and 2.58 (1.04–6.43), respectively).

**Table 3:** Results of univariate and multivariate Cox regression analysis of clinicopathological factors associated with OS and RFS after surgery in the pancreatic adenocarcinoma patients who underwent curative-intent resection after preoperative chemoradiotherapy (n = 141).

				Univariate analysis		Multivariate analysis	
Variable		No. of cases	MST (months)	Hazard ratio (95% CI)	*p*-value	Hazard ratio (95% CI)	*p*-value
**OS after surgery**							
Grade of local invasion before CRT on CT scans	cT1-T3 cT4	64 77	46.1 27.6	1 1.59 (1.05–2.41)	0.030	1 1.66 (1.01–2.73)	0.044
Estimated operative blood loss (x 10^2^ ml)		141	N/A	1.03 (1.02–1.05)	<0.001	1.02 (1.00–1.03)	0.035
Major postoperative complications*	no yes	100 41	42.4 24.5	1 1.64 (1.03–2.54)	0.038		
Conducting adjuvant chemotherapy	yes no	117 24	46.1 8.3	1 3.42 (2.03–5.55)	<0.001	1 3.99 (2.23–7.13)	<0.001
Pathological grade of local invasion	pT1 or T2 pT3	95 46	64.8 27.3	1 2.07 (1.31–3.39)	0.002		
Extrapancreatic nerve plexus invasion	no yes	103 38	44.9 21.5	1 1.78 (1.13–2.73)	0.013		
Degree of the residual tumor	R0 R1	128 13	41.7 19.6	1 2.58 (1.35–4.64)	0.007		
Histological response to CRT	Grade 3 or 4 Grade 1 or 2	63 78	57.8 26.6	1 2.09 (1.35–3.29)	<0.001	1 2.10 (1.29–3.43)	0.003
Peritoneal Washing cytology	CY- CY+	135 6	40.2 9.7	3.51 (1.35–7.50)	0.013	1 5.00 (2.03–12.31)	<0.001
**RFS after surgery**							
Grade of local invasion before CRT on CT scans	cT1-T3 cT4	64 77	27.5 13.3	1 1.69 (1.15–2.50)	0.008	1 1.57 (1.03–2.39)	0.035
Estimated operative blood loss (x 10^2^ ml)		141	N/A	1.02 (1.00–1.03)	0.037		
Conducting adjuvant chemotherapy	yes no	117 24	20.4 9.3	1 2.12 (1.28–3.39)	0.005	1 2.04 (1.18–3.52)	0.011
Pathological grade of local invasion	pT1 or T2 pT3	95 46	32.9 14.1	1 2.13 (1.38–3.38)	0.001	1 1.69 (1.04–2.72)	0.033
Degree of the residual tumor	R0 R1	128 13	19.3 12.7	1 2.57 (1.35–4.50)	0.005		
Histological response to CRT	Grade 3 or 4 Grade 1 or 2	63 78	32.9 14.8	1 1.84 (1.24–2.79)	0.003	1 1.72 (1.10–2.70)	0.018
Peritoneal Washing cytology	CY- CY+	135 6	17.7 8.9	1 2.91 (1.12–6.20)	0.030	1 2.58 (1.04–6.43)	0.041

OS; overall survival, RFS; recurrence free survival, CRT; chemoradiotherapy, CT; computed tomography

* Clavien-Dindo classification IIIa or higher complications.

## Discussion

This study aimed to elucidate the frequency of CY+ and its prognostic significance in patients with localized PDAC who underwent curative-intent resection after preoperative CRT. Additionally, this study aimed to identify preoperative risk factors predicting the occurrence of CY+. Despite the relatively low incidence of CY+ after preoperative CRT (4.3%), it emerged as an independent prognostic factor for worse OS (HR 5.00, 95% CI 2.03–12.31) and RFS (HR 2.58, 95% CI 1.04–6.43), with a significantly elevated prevalence of peritoneal recurrence. The grade of local invasion before CRT on imaging, absence of adjuvant chemotherapy, and limited histological response to CRT were independent predictors of worse OS and RFS. Moreover, the prevalence of tumor location in the body or tail, along with pathological invasion to the anterior pancreatic capsule, was significantly higher in the CY+ group than in the CY− group. Our data support the utility of tumor staging using peritoneal washing CY in patients with localized PDAC who have undergone curative-intent resection after preoperative CRT, particularly in those with potential risk factors associated with CY+.

The observed incidence of CY+ following preoperative CRT in our study cohort was notably low (4.3%), highlighting the potential impact of preoperative CRT in reducing CY+ incidence. Compared with reported rates of CY+ (ranging from 3.9% to 14%) in patients with localized PDAC who underwent upfront surgery, the incidence of CY+ in our cohort was comparatively diminished [15–23]. Moreover, CY+ rates have been shown to correlate with disease stage, with locally advanced PDAC exhibiting a higher incidence of CY+ (typically ranging from 11% to 34%) compared with potentially resectable PDAC [19,24,25]. The current study cohort included borderline resectable or locally advanced PDAC in approximately 70% of the 141 patients who underwent curative-intent resection following preoperative CRT. Nevertheless, the overall CY + rate was comparatively low, suggesting that the preoperative intervention may reduce CY+ rates in patients undergoing subsequent curative-intent resection. A previous investigation analyzing results from staging laparoscopy in 1004 patients with radiographically localized PDAC revealed that individuals subjected to neoadjuvant chemotherapy before laparoscopy exhibited significantly lower rates of positive laparoscopy, including the presence of gross metastases and/or positive peritoneal CY, compared with chemotherapy-naïve patients [26]. Consequently, any administration of preoperative chemotherapy before resection seems to reduce CY+ rates, which is likely attributable to the decreased sensitivity of cytological examination. Despite its low frequency, the current study showed a strong association between CY+ and significantly worse OS (CY+ vs. CY−: 13.3 vs. 40.2 months). Additionally, no significant difference in OS after the initial treatment was observed between the CY+ group and the cohort without resection after preoperative CRT (CY+ vs. no resection: 18.3 vs. 16.3 months, p = 0.868). In a previous study involving patients with potentially resectable or borderline resectable PDAC who underwent surgical resection following neoadjuvant therapy (NAT), predominantly comprising gemcitabine and/or S-1-based chemotherapy, followed by surgical resection, CY+ was associated with significantly shorter OS than CY− (CY+ vs. CY−: 14.8 vs. 30.8 months). CY+ was identified as an independent adverse prognostic factor after subsequent resection. Furthermore, the prognosis of patients with CY+ was not significantly different from that of patients without resection due to macroscopic distant metastasis or extensive local invasion after NAT, which aligns with the results of the survival analysis in the current study [27]. This finding underscores the criticality of assessing peritoneal washing CY even in the context of NAT, including preoperative CRT, because it retains its prognostic significance. Additionally, subsequent resection may not contribute to improved survival in patients with CY+ after preoperative CRT.

This study aimed to identify preoperative risk factors predictive of CY+ occurrence. Tumor location in the body or tail was significantly associated with CY+, implying that the anatomical site of the tumor might influence the likelihood of peritoneal involvement. This information is valuable for risk stratification and treatment planning in patients with PDAC undergoing preoperative CRT. Reported preoperative risk factors predicting CY+ encompass various parameters, including tumor location in the pancreatic body or tail, elevated preoperative serum CA19-9 levels, larger tumor size, and borderline status in tumor resectability [17,22,25,28,29]. In this study, the serum CA19-9 levels before and after CRT were not significantly associated with CY+ occurrence. Considering that surgical indications typically depend on normalization or a substantial decrease in serum CA19-9 levels as criteria for surgical decision-making, CA19-9 may not prove to be a dependable predictor of CY+ in the context of surgery following NAT, as observed in the current study cohort. Consistent with previous findings, tumor location in the pancreatic body or tail was the sole significant factor associated with CY+. A recent investigation involving patients with localized PDAC who underwent staging laparoscopy revealed a relatively high rate of CY+ (20%), even in patients with resectable PDAC. Tumor location in the pancreatic body or tail and tumor size were identified as significant factors predicting CY+ in resectable PDAC, whereas a higher serum DUPAN-2 level was significant in borderline resectable PDAC [25]. In the current study, the incidence of CY+ was significantly higher in pancreatic body or tail cancer than in pancreatic head cancer (head vs. body or tail: 1.9% vs. 11.4%). This finding suggests that tumor staging using peritoneal washing CY must be considered, especially for patients with pancreatic body or tail cancer, even after preoperative CRT.

A comparison of pathological outcomes between the CY+ and CY− groups may reveal several noteworthy findings. However, limited data are available regarding the correlation between CY+ status and other pathological features, particularly in the context of surgery after preoperative CRT. When comparing pathological results, the incidence of anterior pancreatic tissue invasion on the serosal side was significantly higher in the CY+ group than in the CY− group in the current study (66.7% vs. 24.4%, p = 0.041). However, other significant pathological factors, such as extrapancreatic nerve plexus invasion, degree of residual tumor, and histological response to CRT, were not associated with the occurrence of CY. Similarly, previous studies reported a significant correlation between CY+ and tumor invasion of the anterior pancreatic capsule in patients with localized PDAC who underwent upfront surgery [22]. These findings indicate that anterior pancreatic tissue invasion is a crucial pathological factor associated with CY+.

The findings of this study have implications for clinical practice, highlighting the crucial role of incorporating peritoneal washing CY assessment into management strategies for patients with PDAC undergoing preoperative CRT. In this study, the absence of adjuvant chemotherapy emerged as an independent prognostic factor affecting both OS and RFS. Notably, the CY+ group exhibited a high incidence of peritoneal recurrence after surgery. This recurrence was characterized by early tumor regression and a dismal prognosis. Although adjuvant chemotherapy was administered to four out of six patients with confirmed CY+, it did not prevent the observed peritoneal recurrence. Satoi et al. previously investigated the significance of adjuvant chemotherapy in a multicenter study focusing on patients with localized PDAC and confirmed CY+ status undergoing margin-negative resection. The results indicated that adjuvant chemotherapy did not yield favorable survival outcomes in patients with CY+ [30]. Surgical resection following preoperative CRT may impede the seamless initiation of postoperative systemic chemotherapy owing to the occurrence of complications or worse performance status, particularly in surgery for locally advanced PDAC, known as conversion surgery. Therefore, in cases of CY+ PDAC after preoperative CRT, additional and more potent systemic chemotherapy should be considered, even when the local status is surgically resectable. Staging laparoscopy with peritoneal washing CY may be beneficial during the re-evaluation of resectability after preoperative CRT, especially for patients with pancreatic body or tail cancer, as well as for those with elevated preoperative CA19-9 levels, given the significantly higher incidence of CY+ in pancreatic body or tail cancer compared to pancreatic head cancer, and the significantly higher level of preoperative CA19-9 in CY+ group in our study. However, further investigation is necessary to identify the risk factors predicting CY+ and to establish the optimal indications for staging laparoscopy after preoperative CRT.

The current study had several limitations. First, it was a retrospective study conducted at a single institution with a limited number of patients, potentially introducing unknown confounding factors and selection bias. Second, the CY+ cohort included only a limited number of patients due to the rarity of the condition in the CY+ group, resulting in an imbalance between the CY+ and CY- groups exists. However, obtaining a substantial number of patients with CY+ who underwent curative-intent resection following preoperative CRT may prove challenging in a single-institutional study. Third, individuals who did not undergo curative-intent resection, along with those without peritoneal washing CY after preoperative CRT, were excluded from the current study, introducing potential selection bias due to the lack of CY status. To address these limitations, prospective studies with larger cohorts are required to validate these findings. Multi-institutional studies may increase the sample size and fasilitate the exploration of additional risk factors influencing the occurrence of CY+ in the context of preoperative CRT, even in the retrospective settings. Nevertheless, the present study provides crucial insights into the optimal treatment strategy for patients with confirmed CY+ status after preoperative CRT. This is one of the most extensive single-center studies to assess the validity of surgical resection in patients with confirmed CY+ status after preoperative CRT.

## Conclusion

In conclusion, among patients with localized PDAC who underwent curative-intent resection after preoperative CRT, CY+ status was identified as an independent adverse prognostic factor, correlating with a significantly higher incidence of peritoneal recurrence. Notably, the CY+ cohort exhibited a significantly higher incidence of tumor location in the body or tail of the pancreas, along with pathological invasion to the anterior pancreatic capsule, than the CY− group. Our findings underscore the necessity of using peritoneal washing CY to determine the indications for surgical resection, even after preoperative CRT, in patients with localized PDAC, particularly in those with potential risk factors associated with CY+.

## Supporting information

S1 Data set (XLSX)
